# Many Important Items of News

**Published:** 1890-08

**Authors:** 


### Medical News and Miscellany

**Dr. F. H. Tucker**, of San Augustine, is convalescent from a severe illness.

**Pilocarpine**, hypodermically administered, has recently been used with success in cases of chronic articular rheumatism.

**Dr. C. T. Cooper**, who removed from Lilac, Texas, to Boone, Iowa, last September, has returned to Texas and located at Taylor.

**Dr. W.P. Markham** has removed from Decatur to Denison. Also Dr. W. S. Nobles from Collinsville to Denison. The latter is associated with Dr. Nagle in a special practice.

Dr. A. S. McDaniel, formerly of Columbus, Texas, has taken an *ad eundem* degree at Bellvue, and has located at San Antonio.

— —

Our frend Dr. L. B. Creath has pulled up stakes at Kinneyville, and is traveling over the State in search of a better location.

---

Dr. Charles M. Harrison has removed from El Paso to Tampico, Mexico. He sends three years subscription to the JOURNAL, from the land of the Montezumas.

---

Eight thousand physicians attended the Berlin International Medical Congress. Think of 8000 doctors—of many different nationalities—and perhaps all talking at once! Babel was nothing in comparison.

---

Dr. D. C. Jones has removed from Calvert to Waco. Waco seems to be absorbing all the medical men in that section, as we have noted the removal of some half a dozen or more physicians to that city recently, from the central part of the State.

---

Removals.—Dr. A. Sims has removed from McKinney to Weatherford.

Dr. A. D. Borroughs has removed from Lovelady to Houston.

Dr. D. B. McGee has removed from May to Brownwood.

---

State Health Officer Rutherford has made a tour of inspection on the Mexican frontier and in eastern Mexico, and says the reports of small-pox are greatly exaggerated, and that he has an efficient *cordon sanitaire* along the border, which will surely prevent an invasion of Texas soil.

---

Shrady the Wise.—The New York *Medical Record*, in an editorial on the recent Medical Congress at Berlin, says that "only those who have an axe to grind, or those little fellows who do so love to rub up against the big fellows, ever attend medical congresses: the wise men stay at home." Shrady staid at home,—*ergo*—.

**Dr. W. A. Morris**, a venerable and beloved physician of Austin, is convalescent from a spell of dysentery, complicated in its course with peritonitis—in which, at one time, his life was despaired of. He sank and was thought to be dying—being profoundly unconscious; but he was resuscitated by means of a galvanic battery.

---

**The Medical Archives of the C. S. Army.**—Attention is called to the correspondence between ex-Medical Director Stout, C. S. A., and Prof. Joseph Jones, of New Orleans, published elsewhere in this issue. Newspapers friendly to the cause will render a service to ex-Confederate surgeons and the public by copying the letters.

---

**Col. J. H. Baxter**, Chief Medical Purveyor U. S. A., has been appointed Surgeon-General with the rank of Brigade-General. We forgive all of Mr. Harrison's mistakes in consideration of his having the good sense to recognize the eminent fitness and just claims of Dr. Baxter for this high office. The Doctor has the JOURNAL'S sincere congratulations.

---

**Rembrandt's Anatomical Lesson.**—We have received from the Cincinnati Gravure Company a handsome engraving with the above title. It is announced as "the Physicians' and Surgeons' picture, *par excellence;*" price by mail, post-paid, $2. It is printed on heavy plate paper. Size 22x28 inches. With India tint borders and enbossed title. It is a very handsome and highly artistic piece, and the JOURNAL highly appreciates it as a present.

---

**The Ashville Medical Review** is the name of a new journal, to be issued at Ashville, N. C., on the 15th inst., and monthly thereafter. Drs. Frank T. Merriweather and H. Longstreet Taylor are the editors and publishers. Subscription price $2 a year. The object of its publication, as anounced, is to keep the medical profession informed of "the advantages and climate" of Ashville as a health resort, "from a scientific standpoint." We wish the *Review* success.

**The Austin Distrtict Medical Society.**—This flourishing Society will hold its next regular meeting on the 25th of September. A good programme has been prepared, and a full attendance is expected. The following papers have been announced: "Traumatic Tetanus," "Eclampsia," "The Treatment of Epithelioma of Face with Caustics," "Some of the Delusions of the Insane," "Antipyretics," and the Presidential address. Officers will be elected at this meeting for the ensuing year. Every regular physician accessible to Austin is invited to be present and take part in the proceedings.

---

**Creosote in Diabetes.**—Two cases of diabetes have been treated with excellent results by Valentini by means of creosote administered internally. In one case four drops per diem were given at first, this quantity being afterwards increased to ten drops. Under this treatment the sugar disappeared, and did not return when he began to eat starchy food. The other patient was given six drops per diem, and did equally well.—*Lancet, in St. Joseph Med. Herald.*

---

C. L. Hall, M. D., of Marshall, Mo., in the *St. Joseph Medical Herald*, calls attention to the following prescription as an efficient remedy in nasal catarrh:

| | | |
|---|---|---|
| ℞ | Resorcin | gr. xxv. |
| | Aquæ | q. s. |
| | Menthol | gr. iii–v. |
| | Vaselene Alba. | ℥ i. |
| | M. ft. mist. | |

Sig.: Use with an Aloe's ointment atomizer.

---

**Loco-Weed.**—Mary Gage Day, M. D., of Wichita, Kan., in the *New York Medical Journal* of Nov. 30, 1889, gave a process by which she has separated crystals of microscopic size, blue-white in color, and of a variety of forms. The most characteristic are slender and pointed, arranged in rosettes or grouped in various ways. These crystals are soluble in water and very dilute alcohol, very sparingly soluble in strong alcohol, and not at all soluble in chloroform or ether.

A small amount of these crystals were fed to a cat, and produced the characteristic poisonous symptoms,—sleepiness, loss of appetite, retching, diarrhœa, etc.

The process of separating the crystals is as follows: The roots, stems, and leaves, gathered in the autumn, were boiled ten hours, strained, and the decoction concentrated to a syrup, poured, while hot, into a hot flask, corked, and set away. At the end of ten days the syrup had separated into two layers, the upper, a blackish liquid, the lower, a brownish sediment. The liquid was poured into a flask and covered with six times its volume of very dilute alcohol (30%), corked, and let stand for three days; agitated occasionally; then filtered, and the filtrate slowly evaporated in the air, when crystals formed. It was found important not to hurry the evaporation, for, when this was done, the crystals did not form.

---

**Abortion of Fœtuses Retained in the Uterus Seven Months**—Dr. E. J. Kempf, Jasper, Ind., in *The North Carolina Medical Journal* for July, 1890, reports an interesting case of twin fœtuses that remained dead in the uterus about seven months. Patient missed the first time in April. She saw nothing until August, when she fell and hurt herself. There was a little flow at the time which continued to recur about the 25th of each month until February. The patient had considered herself pregnant up to August, when the menses began. The patient was advised to take good care of herself, keep the bowels open and wait for developments, as there was no urgent indications for interference. Finally, after taking 1-5 grain of aloin and 1-30 grain strychnine for constipation, a bag of water containing two fœtuses escaped intact—*not a drop of blood.* No irrigation employed. Patient made a rapid and perfect recovery.

This case illustrates the point that one should not be too hasty in interference as long as there are no symptoms demanding that something be done.

---

**Treatment of Peritonitis.**—Lanphear, of Kansas City, in an article (*Medical Review*, July 12, 1890) favoring the cathartic treatment of peritonitis, says that opium possesses, really, but little curative power over the inflammatory process; that it retains within the bowels irritating fæcal matter that can but increase the trouble; that by completely checking peristaltic movements it leads to the formation of adhesions; that it stops excretion and so prohibits the elimination of the poisonous products of inflammation; and that by benumbing sensibility it gives the physician a feeling of (false) security, which is undesirable. Upon the contrary, the administration of the saline cathartic has the power of arresting the disease if given in the stage of invasion; here the peritoneum is simply congested, or just taking on an inflammatory action—the pain is not very severe, the abdomen is not greatly distended, the fever is not high; but the physician is called, recognizes the danger, and immediately gives the saline. If it be the initial stage of a simple peritonitis, if it be the beginning of a puerperal peritonitis, or if it be the sthenic stage of a septic peritonitis, the indication for the cathartic is clear: in either instance the blood vessels are turgid with blood—the circulation impeded; the taking away of a considerable amount of serum from the abdominal viscera must profoundly affect the peritoneal circulation and assist in restoring the normal state. Besides, the active peristaltic action prevents the formation of bands and adhesions if the inflammation goes on; and experience at the bedside demonstrates that as the inflamed surface is relieved from engorgement by the emptying of the intestinal vessels, the temperature and the pulse rapidly decline, while the pain disappears almost as speedily as under opium. If the disease has gone on to the second stage where the fever is high, the pain intense, the tympanites marked and the pulse rapid and wiry, cathartics are not so strongly indicated, though small doses of sulphate of magnesia and tincture of belladonna may often be given with advantage; here the danger is collapse, and as vital force must be conserved, violent purgation is not justifiable, and opium must be given—not to check peristalsis, nor to "put the bowels in splints," but to deaden the pain in the many terminal nerves of the peritoneum, to sustain the heart and to prevent shock. The bowel must be kept free from irritating fæcal matter, by enemata if possible, by a good cathartic if necessary; tympanites must be controlled by turpentine, either internally or by stupes; and the strength must be maintained by milk, whisky, etc. His conclusions are:

1. The saline treatment should be adopted early in simple, acute peritonitis.
2. Small doses of calomel may be given to mild purgation in cases seen after the disease is fully developed.
3. Cases which fail to be relieved by cathartic measures should receive early operative interference.
4. Whenever peritonitis has gone on to that stage where the formation of pus is known, or even suspected, to have taken place, abdominal section and drainage are imperatively indicated.
5. When the existence of tubercular peritonitis is diagnosticated, or strongly suspected, operation (exploring incision) is justifiable.
6. Opium is only indicated in the second stage of peritonitis, and then not because it "forms a splint," but because it relieves pain, sustains the heart and prevents shock—thus combatting the tendency to death.

